# Practices of Adults in a Periurban Community of the Ho Municipality on Prevention of Hypertension

**DOI:** 10.1155/2020/2136213

**Published:** 2020-01-09

**Authors:** Kennedy Diema Konlan, Elizabeth Aku Baku, Milipaak Japiong, Kennedy Dodam Konlan, Abdul Razak Doat, Amos Nawunimali Suuk, Roberta Mensima Amoah

**Affiliations:** ^1^University of Health and Allied Sciences, School of Nursing and Midwifery, Department of Public Health Nursing, Ho, Ghana; ^2^University of Health and Allied Sciences, School of Nursing and Midwifery, Department of Nursing, PMB 31, Ho, Ghana; ^3^Department of Nursing, West End University College, Accra, Ghana; ^4^Tamale Nursing and Midwifery Training College, Tamale, Northern Region, Ghana; ^5^Nursing and Midwifery Training College, Box SL 98, Kpembe, Savana Region, Ghana; ^6^Department of Public Health, School of Allied Sciences, University for Development Studies, Tamale, Northern Region, Ghana

## Abstract

**Background:**

Hypertension remains a rising health threat among developing countries and it is due to poor knowledge and lifestyles. Integrated knowledge and practices are central towards the control of hypertension, especially in the developing world.

**Objectives:**

This study assessed the practices of adults in a periurban community in the Ho Municipality of the Volta region on the prevention of hypertension. *Methodology*. A cross-sectional descriptive research design was carried out in a periurban community in the Volta region. Adults were recruited using a systematic sampling technique in the Ahoe community. Pretested questionnaires were used to collect information on sociodemographic, knowledge, and lifestyle practices regarding hypertension prevention. The data were entered into Microsoft excel 2013 spreadsheet, cleaned, and transported to the Statistical Package for Social Sciences (SPSS) version 22 software for analyses. The data were analysed using simple descriptive statistics.

**Results:**

In this study, 49.3% explained that hypertension means the increased force of blood through the blood vessels as 90.8% indicated that taking antihypertensive medications can control hypertension. In describing the associated effects of hypertension, adults in the Ahoe community described the effects of hypertension as stroke (89.8%), heart attack (84.0%), diabetes (56.5%), and heart failure (82.3%). Also, 54.1% described hypertension as a lifelong disease while 55.8% indicated hypertension can be cured. Also, 92.2% identified exercising as an important factor in controlling hypertension as 32.7% use herbal preparations to control hypertension. Only 3.7% of adults in the Ahoe community were smokers and 54.5% smoked less than a year. The majority (61.6%) of the respondents did not drink alcohol as 69.7% engage in active exercises.

**Conclusion:**

Education on hypertension should be intensified, and emphasis should be laid on regular exercised and strict dietary restrictions that ensure reduction in hypertension risk. Healthcare authorities should engage hypertensive clients to desist from the intake of herbal medications whose actual composition has not been scientifically determined.

## 1. Introduction

The World Health Organization (WHO) has estimated that high blood pressure causes one in every eight deaths, making hypertension the third leading killer in the world [1]. Globally, there are one billion hypertensive patients and four million people die annually as a direct result of hypertension [2]. Hypertension in the local palace in Ghana is regarded as a silent killer because it does not show signs and symptoms in its initial stages. Ghanaians still believed that once it did not lead to health problems in an individual, it was normal while others think hypertension was “*Mogya mbordo*” which means having too much blood [3]. Several factors may be associated with the high spate of hypertension in the country. Addo et al. [4] reported that the high rate of hypertension was associated with low levels of awareness, inadequate drug treatment, and poor blood pressure control. They further reported that overweight and obesity are modifiable risk factors for hypertension that can be addressed through lifestyle interventions. There can be health improvement by integrating hypertension care into primary care in rural health facilities [4]. The level of knowledge demonstrated by persons on their diagnosis may have a useful influence on the treatment, practices, and the lifestyle they adopt. Addo et al. [4] demonstrated that 33% of hypertensive patients in Ghana were aware of their diagnosis, 18% were treated, and 4% had adequate blood pressure (BP) control. Possible explanations for these low numbers may reflect the complex relationship between patients, providers, and economic resources in this part of the world. For example, patients may lack knowledge about hypertension, or they may harbour beliefs that are discordant with those of the traditional medical paradigm regarding the treatment and causes of hypertension [5].

The prevalence of hypertension in Ghana ranges from 19% to 48% [4]. Factors independently associated with hypertension include older age group, overnutrition, and alcohol consumption [4]. Whereas there was a trend towards improved awareness, treatment, and control between 1972 and 2005, less than one-third of hypertensive subjects were aware they had hypertension and less than one-tenth had their blood pressures controlled in most studies [3, 4, 6]. Hypertension is clearly an important public health problem in Ghana, even in the poorest rural communities. Opportunities such as the National Health Insurance Scheme (NHIS), a health policy emphasizing health promotion and healthier lifestyles and effective treatment, should help prevent and control hypertension [6], yet hypertension still remains a leading reason for all outpatient attendances in health facilities. Due to the high morbidity and mortality rate associated with hypertension, it has become imperative to assess the knowledge and lifestyle practices pertaining to hypertension among adults living in periurban communities. Current studies have identified that up to 70% of persons identified to have hypertension are not on treatment and only 13% of those with hypertension have their blood pressures well controlled. The prevalence of adult diabetes in Ghana is about 9%, comorbidity to hypertension. The risk factors for developing NCDs are worsening [7]. The United Nations reported that sub-Saharan Africa is rapidly undergoing urbanization with the migration of persons from rural to urban areas [8] and this will likely be accompanied by changes in lifestyle. In addition, the improvements in the economies and standards of living will lead to an increase in life expectancy on the African continent which will also contribute to the increase in the burden of NCDs including hypertension.

Ghana is recently gaining a lower-middle-income status, and the attendant challenges of middle-income countries are inevitable with increasing rural and urban migration and sprouting of periurban communities. There is also an associated increase in livelihood with its associated sedentary lifestyle, increasing the risk of cardiovascular diseases which are linked to a sedentary lifestyle [9, 10]. This also leads to an increase in social vices like smoking [11, 12] and consumption of a high-fat diet [13, 14] further deepening the hypertension burden.

In most periurban communities, apart from known hypertensive patients who have some knowledge about the condition and some lifestyle restrictions or modifications prescribed for them by the healthcare team, the general public is not well acquainted with this. Lifestyle can only be modified by an individual's own decision based on his level of knowledge. Therefore, this study assessed the knowledge and lifestyle practices pertaining to hypertension of the inhabitants of the Ahoe community in the Ho Municipality.

## 2. Objective

This study assessed the practices of adults in a periurban community in the Ho Municipality of the Volta region on the prevention of hypertension.

## 3. Methodology

### 3.1. Study Design

This study used a descriptive cross-sectional study design. People above 18 years in the Ahoe community of the Ho Municipality were contacted to respond to a questionnaire with no follow-up required.

### 3.2. Setting

The Ho Municipality, one of the municipalities in the Volta region, was established by a Legislative Instrument (LI) 2074 of 2012. Originally, Agotime Ziope and Ho West were all part of the then Ho District until 2012 when these districts were carved out. The Municipality has Ho as its capital, which also serves as the capital and economic hub of the Volta region. The population of the Ho Municipality according to the 2010 Population and Housing Census is 177,281 representing 8.4 percent of the region's total population. Females constitute 52.7% (93,469) and males represent 47.3% (83,819) [15]. About 62.1% (110,048 persons) of the population reside in urban localities as compared to 37.9% (67,233 persons) in the rural areas [15]. The youthful population in the Municipality accounts for 31% of the population with a small number of elderly people. According to the HMA [16], Ahoe is not a typical slum, but it is a rundown residential area together with three others in Ho: Bankoe, Hliha, and Dome. It is estimated that 36% of the population of Ho live in these rundown residential areas. Ahoe has a population size of 1,234 [16] and has limited access routes and community facilities. Hypertension is one of the top three leading causes of all deaths and among the top ten reasons for outpatient attendance in the Volta regional [19] and the Ho municipal hospitals. The Ho teaching hospital (formally called the Volta regional hospital) and the Ho municipal hospitals principally serve the Ahoe and adjourning periurban communities. These two health facilities have consistently reported an increase in threat of the number of hypertensive patients. There are about six periurban communities within the Ho township. These periurban communities include Ahoe, Dome, Banko, Barracks Newtown, Deme/zongo, and Lokkoe [16]. Among these communities, the Ahoe community was selected based on random sampling method.

### 3.3. Study Population

The target population for this study was all adult residents in the Ahoe community of the Ho Municipality—a periurban community. According to the United Nations Human Settlements Programme, Ghana Ho City Profile (2009), Ahoe has a population size of 1,234 [16]. The Ahoe community can be divided into five strata. Using the Ahoe roundabout as a central point, the four roads that meet at this point allow the community to be divided into four strata while the area around the Assemblies of God church was considered as the fifth strata. The total number of households in the Ahoe community is estimated to be at about 1600 households. Only respondents who were at the period of the study residents of the Ahoe community were recruited into the study.

### 3.4. Sample and Sampling Technique

The required sample size was calculated using Yamane's formula for calculating sample of known population size [20]:

(1) $$\begin{array}{c} n=\frac{N}{1+Ne^{2}}, \end{array}$$

where *n* is the sample size, *N* is the population size which is 1,234 [16], and *e* is the margin of error which is set at 0.05.

A sample size of 310 was extrapolated.

The respondents were sampled using systematic sampling methods. The sampling fraction was calculated to be 5. At a central point in each stratum within the community, five research teams were dispatched in five different directions. Each research team of two people was to administer the questionnaire in every fifth household. The respondents were recruited from their homes for the study. The study took place from half-past six to half-past five within a one-week interval. Respondents who consented to take part in the study were recruited. The study took place in April 2019. Prior to the commencement of data collection, five research assistants who assisted in the data collection had a day's training in quantitative data collection.

### 3.5. Data Collection and Analysis

A pretested questionnaire was used as the data collection instrument. Prior to the collection of the data, the study tool was pretested in the Bankoe community. The Bankoe community share boundaries with the Ahoe community and have the same demographic characteristics as the Ahoe community. The pretesting assisted in identifying ambiguity and also the appropriateness of the questions in the study tool. Pretesting also assisted in the utilisation of more appropriate words in the local languages when interpretation of the questionnaire in the local languages was required. The questionnaire was modified until it produced a *Cronbach alpha coefficient* of 0.811. It can, therefore, be concluded that the instrument had a high reliability in measuring the needed data for the study. Responses from the people showed that the questionnaire was clear and could be understood by others.

The questionnaire was divided into sections: demographic data, knowledge levels on hypertension, hypertension status, and lifestyle practices and treatment modalities used by hypertension patients. On each day of the data collection, research assistants together with the researchers in a team reviewed each questionnaire for appropriateness of response and subsequently coded the same for data entry. Using a systematic sampling method, 16 (5.1%) of the 310 persons sampled did not consent or withdrew from the study before the completion of the questionnaire. This questionnaire which did not meet the minimum requirement for entry was discarded. Reasons for withdrawal were that they had a busy schedule and could not stay long enough to complete a questionnaire. The data were entered into Microsoft excel 2013 spreadsheet, cleaned, and subsequently transferred to the Statistical Package for Social Sciences (SPSS) version 22.0 for analysis. Data were analysed using descriptive statistics. In total, 294 questionnaires met the criteria for and were used in the data analysis.

### 3.6. Ethical Considerations

The study received scientific approval from the School of Nursing and Midwifery Research and Scientific Committee of the University of Health and Allied Sciences. Also from the Institute of Health Research (IHR), University of Health and Allied Sciences, Ho, ethical approval was obtained to conduct this study (UHAS–REC A.2 [20] 18-19). A formal permit was secured from opinion leaders of the Ahoe Community prior to the commencement of the study. The purpose and objectives of the study were explained to each respondent. Respondents' privacy, confidentiality, and anonymity were ensured throughout the collection, handling, and usage of the data.

## 4. Results

As shown in Table 1, more than half (55.4%) of the respondents were male. The mean age with standard deviation was 34.09 ± 13.97 with modal age (53.7%) between 20 and 29 years. The majority (47.6%) of the respondents had attained secondary education. A greater proportion (96.6%) were Christians.

Adults in the Ahoe community (49%) described hypertension as an increased force of blood through the blood vessels. The majority (97.3%) of the respondents indicated normal systolic blood pressure as less than 120 mmHg as 74.1% indicated normal diastolic pressure as less than 80 mmHg. Respondents (69.0%) stated one of the symptoms of hypertension as difficulty in breathing (Table 2).

As shown in Table 3, in describing associated causes of hypertension, adults in the Ahoe community (89.8%, 84.0%, 56.5%, and 82.3%) described the causes of hypertension as stroke, heart attack, diabetes, and heart failure, respectively. Also, 54.1% described hypertension as a lifelong disease while 55.8% indicated hypertension can be cured. The majority of the respondents indicated that hypertension can be prevented through taking antihypertensive medications (90.8%), exercising (92.2%), losing of excessive weight (84.7%), having less stress (85.0%), and avoiding smoking (92.2%). The majority (85.0%) of adults in the study area indicated that changing unhealthy diet with healthy diet helps in the prevention of hypertension. Most (86.7%) of the respondents indicated reducing alcohol intake help in controlling hypertension. The majority (77.2%, 81.6%, 81.3%, 74.5%, and 53.1%) reported that excessive weight gain, excessive intake of alcohol, excessive smoking, excessive salt intake, and ageing were risk factors of hypertension, respectively.

A proportion (18.7%) of adults in the Ahoe community were known hypertensive. The majority (74.5%) of the respondents were diagnosed more than a year ago as 81.1% of the hypertensive patients were on antihypertensive drugs, but only 64.4% were taking the antihypertensive drugs at the time of the study. Among the 35.6% of hypertensive who did not take the antihypertensive drugs, 43.7% gave reasons that they were tired of taking the drugs. Some of the hypertensive patients (41.8%) could not remember the last time they checked their blood pressure as shown in Table 4.

From Table 4, the majority (67.3%) of hypertensive patients did not take herbal medicine. About 44% have been taking herbal medication since diagnosis as 55.6% took herbal medication with orthodox medication for treating hypertension. Some hypertensive patients (38.9%) have informed their doctors of the fact that they were taking herbal medicine as well. Describing their perception of improvement since taking herbal medication, 66.7% indicated it has been helpful. The findings also showed that 75.0% of hypertensive patients who sought treatment from prayer camps did not experience an improvement. The majority (92.2%) of the respondents indicated that lifestyle changes prevent hypertension. The majority (94.9%) of the respondents were ready to change their lifestyle to prevent hypertension. Only 3.7% of adults in the Ahoe community were smokers and 54.5% smoked less than a year. The majority (61.6%) of the respondents did not drink alcohol as 69.7% engage in active exercises.

## 5. Discussion

This study assessed the practices of adults in a periurban community in the Ho Municipality of the Volta region on the prevention of hypertension. It was identified that 18.7% of respondents were known hypertensive. This was generally more than the reports of other studies on the prevalence of hypertension. Sanuade et al. [19] reported that the overall prevalence of hypertension in the Ghanaian population is 13.0%. Central to the prevalence of hypertension is population-based studies where individuals will independently indicate their status. The Ahoe community, increasingly becoming a periurban community, members are daily engaged in activities that increase hypertension risk. Most of the respondents (74.5%) who indicated they were hypertensive were diagnosed over a year ago. The community saw a rapid growth within the last decade and might be related to the spate of the disease among inhabitants.

Adults in the Ahoe community (49.3%) correctly explained that hypertension means the increased force of blood through the arteries. The level of knowledge of hypertension is divided among the adult population and likely to influence the activities they adopt to prevent the disease. Improved knowledge on a particular habit is likely to lead to an improvement in behaviours that reduce the risk. Anowie and Darkwa [3] reported that more than half of the respondents (63.8%) correctly explained that hypertension occurred when one's blood pressure moved higher than normal. The majority of adults in the Ahoe community were aware of the normal systolic and diastolic blood pressure levels. In this study, 97.3% and 74.1% indicated normal systolic and diastolic blood pressure levels were less than 120 mmHg and 80 mmHg, respectively. Kisokanth et al. [20] reported that around 42.7% stated that 120/80 mmHg was the normal blood pressure. This knowledge on blood pressure limits affords community members to be able to interpret the results of blood pressure checked. Improvement in understanding the course of the disease leads to improvement in adapting activities that prevent the disease. Adults (56.5%) identified that there is a relationship between diabetes and hypertension. Ferrannini and Cushman [21] agreed to this relationship by stating that high blood pressure is reported in over two-thirds of patients with type 2 diabetes. Also, 82.3% identified that hypertension can lead to heart failure and 89.8% indicated stroke. The people of Ahoe have knowledge of some of the complications of hypertension. Rahman et al. [22] reported that over 50.0% of the respondents were aware of hypertension leading to the development of stroke, heart attack, and heart failure. Also, hypertension is reported to be a significant risk factor for the development of congestive heart failure [23].

Various lifestyle changes are needed for the prevention of hypertension. Adults in the Ahoe community of the Ho Municipality identified that hypertension can be prevented through taking antihypertensive medications (90.8%), exercising (92.2%), losing excessive weight (84.7%), having less stress (85.0%), avoiding smoking (92.2%), changing unhealthy diet with healthy diet (85.4%), and reducing alcohol intake (86.7%). There are many underlying factors associated with the occurrence of hypertension. These factors include aging, excessive salt intake, sedentary lifestyle, smoking, alcohol intake, overweight, and obesity as well as genetic factors [24]. Identification of these factors is the first step towards instituting mitigating factors as studies in other jurisdictions produce similar findings. Dejene-Daniel and Kamal [25] reported that 82.18% believe that it is good to avoid extra added salt in their diet. Anthony et al. [26] reported that more than 96.0% of the respondents believe that avoiding extra salt was associated with nonmedical management (NMM) of hypertension. Kisokanth et al. [20] showed that 56% of the respondents knew the benefit of stress reduction as NMM of hypertension as Khan et al. [27] reported that 44.1% reducing stress is one of the NMM for hypertension. A large proportion (81.6%) of the respondents recorded excessive intake of alcohol, as a risk factor for developing hypertension. Mittal and Singh [28] reported that high consumption of alcohol has been related to the rise of blood pressure over the years. This is due to the fact that the kidney and liver work extra hard at getting rid of waste from the bloodstream; therefore, more pressure is exerted on the arteries. Excessive alcohol intake can also increase the chance of other medical issues as obesity that may lead to an increase in blood pressure. The majority (81.3%) of adults in the Ahoe community indicated that excessive smoking leads to the development of hypertension. Kisokanth et al. [20] reported that most of the respondents stated that stress, high salt intake, high intake of alcohol, and smoking were aggravating factors for hypertension.

The population is aware of the benefits of antihypertensive drugs (90.8%) in controlling increased blood pressure; hence, it will encourage them to take their medications. To control blood pressure, adherence with antihypertensive medications or taking medications as prescribed is essential [1]. According to the WHO [2], adherence was defined as the extent to which a person's behaviours (taking medication, following a diet, and/or executing lifestyle changes) corresponds with agreed recommendations from a healthcare provider. The population is knowledgeable about the benefits of exercise (92.2%) in controlling hypertension and will take the initiative to maintain a habit of exercising. JNC VII [29] recommended that hypertensive patients who have the capacity to do aerobic physical exercise should engage in regular aerobic physical activity at least 30 minutes per day and most days of the week. Regular aerobic physical activity (e.g., brisk walking) at least 30 minutes a day most days of the week and moderately intense activity such as brisk walking, jogging, and swimming can lower BP, promote relaxation, and decrease or control body weight. There is an increasing likelihood that blood pressure will be controlled amongst the majority of hypertensive patients because they consistently took their medications (64.4%). WHO [2] recommends good adherence to taking medications as a panacea to hypertension control. This has been associated with improved BP control and reduced complications of hypertension [1]. Ramli et al. [30] indicated that poor medication adherence was found to negatively affect blood pressure control.

Some (32.7%) hypertensive clients in the community use herbal medications to control hypertension. There is a deep-rooted belief in plants' potency in hypertension control or management. Humidat and Khamaysa [31] posited that an appreciable number of the population still uses herbal medicine, indicating a deep-rooted belief in the healing potential of plants in Palestine. The efficacy of herbal medications in most instances could not be independently verified as its composition was not determined. Also, 44.4% take herbal medication together with other medications prescribed in orthodox facilities. This increases the likelihood of possible drug-herb interactions, which can produce fatal effects. This phenomenon is not uncommon as herbal medicines were often coadministered with allopathic agents in hypertensive patients, and healthcare givers need to be vigilant and include questions about the use of herbal medicines when taking a patient's drug medication history [32]. Morgan and Watkins [33] posited that herbal remedies are often taken in addition to prescribed drugs in hypertension control.

Almost all (98.0%) of the respondents knew about the importance of a healthy lifestyle in the prevention of hypertension with 94.0% of respondents expressing their willingness to change their lifestyle if needed to control or prevent hypertension. With this level of awareness and willingness to change to prevent or control hypertension, it can be implied that much commitment will be shown in employing healthy lifestyle activities to prevent or control hypertension. Dickey and Janick [34] postulated that there is ample evidence that supports the beneficial effects of healthy lifestyle modifications in the prevention and management of hypertension.

## 6. Conclusion

Education on hypertension and use of behaviour change communication strategies should be intensified as the emphasis is laid on people maintaining healthier lifestyle practices to prevent or control hypertension. Healthcare providers should give much attention to educating and encouraging hypertensive patients to adhere to treatment regimen to ensure that such patients continually take their medication and adopt healthier lifestyle practices. These lifestyle modifications should focus on exercise and adequate dietary restrictions. There should also be increased dialogue between the healthcare team and patients to include assessment of the patient's herbal preparation usage in concomitance with prescribed allopathic agents. Hypertensive patients should be advised to desist from taking herbal preparations whose composition cannot be verified by healthcare regulation authorities.

## Figures and Tables

**Table 1:** Sociodemographic characteristics.

Variables	Parameter	Frequency	Percentage
	Total	294	100.0
	**Age**	**34.09** ± **13.97**	
Gender	Female	131	44.6
	Male	163	55.4
Age	20–29	158	53.7
	30–39	44	15.0
	40–49	25	8.5
	50–60	67	22.8
Educational level	Basic	64	21.8
	No formal education	9	3.1
	Secondary	140	47.6
	Tertiary	81	27.6
Religion	Christianity	284	96.6
	Islam	8	2.7
	Traditionalist	2	0.7

**Table 2:** Definition and symptoms of hypertension.

Variables	Parameter	Frequency	Percentage
Meaning of hypertension	High level of stress	62	21.1
	Rapid pulse	20	6.8
	Increased force of blood	145	49.3
	Do not know	67	22.8

Normal systolic blood pressure	Less than 120 mmHg	286	97.3
	More than 120 mmHg	8	2.7

Normal diastolic blood pressure	Less than 80 mmHg	218	74.1
	More than 80 mmHg	76	25.9

Symptoms of hypertension	Cannot breathe	203	69.0
	Do not know	18	6.1
	Headache, dizziness, tiredness	73	24.8

**Table 3:** Knowledge of factors associated with hypertension.

Variables	Parameters	Frequency	Percentage
Hypertension causes stroke	No	30	10.2
	Yes	264	89.8
Hypertension causes heart attack	No	47	16.0
	Yes	247	84.0
Hypertension causes diabetes	No	128	43.5
	Yes	166	56.5
Hypertension causes heart failure	No	52	17.7
	Yes	242	82.3
Hypertension is a lifelong disease	No	135	45.9
	Yes	159	54.1
Hypertension can be cured	No	130	44.2
	Yes	164	55.8
Taking medications controls hypertension	No	27	9.2
	Yes	267	90.8
Exercising controls hypertension	No	23	7.8
	Yes	271	92.2
Losing weight controls hypertension	No	45	15.3
	Yes	249	84.7
Less stress controls hypertension	No	44	15.0
	Yes	250	85.0
Quitting smoking controls hypertension	No	23	7.8
	Yes	271	92.2
Excessive weight gain causes hypertension	No	67	22.8
	Yes	227	77.2
Excessive alcohol consumption causes hypertension	No	54	18.4
	Yes	240	81.6
Smoking causes hypertension	No	55	18.7
	Yes	239	81.3
Excessive salt intake causes hypertension	No	75	25.5
	Yes	219	74.5
Ageing causes hypertension	No	138	46.9
	Yes	156	53.1

**Table 4:** Lifestyle practices related to hypertension.

Variables	Parameter	Frequency	Percentage
Known hypertensive	No	239	81.3
	Yes	55	18.7
When diagnosed	More than a month	6	10.9
	Less than a year	8	14.5
	More than a year	41	74.5
On medication	No	10	18.2
	Yes	45	81.8
Taking medication	No	16	35.6
	Yes	29	64.4
Reasons for drug noncompliance	Cannot afford medication	6	37.5
	Medications are bitter	3	18.8
	Tired of taking them	7	43.7
Last time checked blood pressure	Less than a month	17	30.9
	Less than a year	15	27.3
	Do not remember	23	41.8
Taking herbal medication	No	37	67.3
	Yes	18	32.7
Duration of taking herbal preparation	About a month ago	5	27.8
	Since diagnoses	8	44.4
	About a year ago	5	27.8
Taking herbal medication with prescribed drugs	No	8	44.4
	Yes	10	55.6
Informed doctor on herbal medication use	No	11	61.1
	Yes	7	38.9
Improvement since taking herbal medication	Yes, witnessed significant improvement	12	66.7
	Yes, fair improvement	5	27.8
	No improvement	1	5.6
Seeking cure through prayer camps	No	290	98.6
	Yes	4	1.4
Outcome of seeking a cure from prayer camps	Was cured	1	25.0
	Nothing changed	3	75.0
Lifestyle changes to prevent hypertension	No	23	7.8
	Yes	271	92.2
Healthy lifestyle important for preventing illness	No	6	2.0
	Yes	288	98.0
Changing lifestyle to prevent hypertension	No	15	5.1
	Yes	279	94.9
Smoking habits	Yes	11	3.7
	No	283	96.3
Duration of smoking	Less than a year	6	54.5
	3-4 years	1	9.1
	More than 4 years	4	36.4
Alcohol use	No	181	61.6
	Yes	113	38.4
Frequently exercised	No	89	30.3
	Yes	205	69.7
Frequency of exercise	Occasionally	125	61.0
	Often	79	38.5
	Not at all	1	.5
Eating late in the night	Yes, occasionally	113	38.4
	Yes, often	74	25.2
	No, not all	107	36.4

## Data Availability

All datasets from which the conclusion of this manuscript is based have all been stated in this manuscript and there are no data deposited in any data repositories elsewhere.
